# Pseudorabies virus exploits N^6^-methyladenosine modification to promote viral replication

**DOI:** 10.3389/fmicb.2023.1087484

**Published:** 2023-02-03

**Authors:** Pei-Lun Yu, Rui Wu, San-Jie Cao, Yi-Ping Wen, Xiao-Bo Huang, Shan Zhao, Yi-Fei Lang, Qin Zhao, Ju-Chun Lin, Sen-Yan Du, Shu-Min Yu, Qi-Gui Yan

**Affiliations:** ^1^Department of Preventive Veterinary Medicine, Swine Disease Research Center, College of Veterinary Medicine, Sichuan Agricultural University, Chengdu, China; ^2^Key Laboratory of Animal Disease and Human Health of Sichuan Province, Sichuan Agricultural University, Chengdu, China

**Keywords:** pseudorabies virus, N^6^-methyladenosine, replication, regulation, m^6^A regulators

## Abstract

**Introduction:**

Pseudorabies virus (PRV) is the pathogenic virus of porcine pseudorabies (PR), belonging to the *Herpesviridae* family. PRV has a wide range of hosts and in recent years has also been reported to infect humans. N^6^-methyladenosine (m^6^A) modification is the major pathway of RNA post-transcriptional modification. Whether m^6^A modification participates in the regulation of PRV replication is unknown.

**Methods:**

Here, we investigated that the m^6^A modification was abundant in the PRV transcripts and PRV infection affected the epitranscriptome of host cells. Knockdown of cellular m^6^A methyltransferases METTL3 and METTL14 and the specific binding proteins YTHDF2 and YTHDF3 inhibited PRV replication, while silencing of demethylase ALKBH5 promoted PRV output. The overexpression of METTL14 induced more efficient virus proliferation in PRV-infected PK15 cells. Inhibition of m^6^A modification by 3-deazaadenosine (3-DAA), a m^6^A modification inhibitor, could significantly reduce viral replication.

**Results and Discussion:**

Taken together, m^6^A modification played a positive role in the regulation of PRV replication and gene expression. Our research revealed m^6^A modification sites in PRV transcripts and determined that m^6^A modification dynamically mediated the interaction between PRV and host.

## 1. Introduction

Pseudorabies (PR) is an infectious disease caused by pseudorabies virus (PRV), which is classically known as Suid herpesvirus I and belongs to the *Herpesviridae* family (Pomeranz et al., 2005). The virus mainly infects suckling piglets within 3 weeks, with symptoms such as elevated body temperature, dyspnea, eyelid edema, gastrointestinal, and neurological symptoms (Rziha et al., 1986). The first case of human endophthalmitis caused by PRV infection was reported in 2017 in China (Ai et al., 2018). Since the discovery in 2020 that PRV infection can also cause acute encephalitis in humans (Liu et al., 2020), there have been more and more studies on PRV. PRV consists of single-molecule double-stranded linear DNA (Roizman and Pellett, 2001), and the genome contains at least 70 genes encoding approximately 100 proteins (Mettenleiter, 2000). PRV proteins are involved in many viral infection processes, and identifying additional mechanisms that regulate viral replication is essential for understanding the life cycle of PRV.

N^6^-methyladenosine (m^6^A) modification is the most common RNA methylation modification in eukaryotic cells. Desrosiers et al. (1974) firstly identified the presence of m^6^A modification in Novikov hepatoma cell mRNAs. m^6^A modification is typically enriched in the 3′ untranslated region (3′ UTR), and low levels are found in the 5′ UTR and the 5′ end of the coding sequence (CDS; Asada et al., 2020). m^6^A modification is a dynamic process co-regulated by methyltransferase complex “writers,” demethylases “erasers” and specific binding proteins “readers.” m^6^A methylation is catalyzed by a large methyltransferase complex composed of writers and other subunits, such as methyltransferase-like 3/14/16 (METTL3/14/16; Liu et al., 2014; Su et al., 2022), Wilms tumor 1-associated protein (WTAP; Ping et al., 2014), vir-like m^6^A methyltransferase associated (VIRMA; Yue et al., 2018), RNA-binding motif protein 15/15B (RBM15/15B; Patil et al., 2016), and so on. METTL14, as the core subunit, forms heterodimer with METTL3 and catalyzes m^6^A modification (Gao et al., 2021). WTAP, as the regulatory subunit, recruits METTL3 and METTL14 into nuclear speckles (Oerum et al., 2021). VIRMA can recruit METTL3 and METTL14, while RBM15/15B can direct METTL3-METTL14 heterodimer to specific RNA sites (An and Duan, 2022). Demethylases erasers include fat mass and obesity-associated protein (FTO) and alkB homolog 5 (ALKBH5), which affect mRNA nuclear export and RNA metabolism (Zheng et al., 2013). ALKBH5 significantly regulates the metabolism and gene expression, and has extensive biological effects on m^6^A modification (Wang and Zhou, 2022). In addition, m^6^A modification sites can be recognized by specific binding proteins readers, of which proteins containing YTH domain are the most studied (Zhao et al., 2020). YTHDF1 regulates mRNA stability and promotes mRNA translation (Wang et al., 2015). YTHDF2 promotes mRNA degradation (Du et al., 2016). YTHDF3 functions as both YTHDF1 promoting translation and YTHDF2 promoting degradation (Li et al., 2017; Shi et al., 2017). Although all act as readers of m^6^A modification, some studies involving YTHDFs present conflicting results (Yu et al., 2021). Moreover, m^6^A modification promotes translation or affects the stability of transcripts depending on which readers exist or dominate under the specific cellular environment (Shi et al., 2019) and the activity of readers that bind different m^6^A sites determines the increase or decrease in mRNA stability (He and He, 2021). In addition to YTHDFs, YTHDC1/2 (Xiao et al., 2016), ELAV-like RNA-binding protein 1 (ELAVL1; Fan and Steitz, 1998; Salton et al., 2011), heterogeneous nuclear ribonucleoprotein A2/B1 (HNRNPA2B1; Alarcón et al., 2015), insulin-like growth factor 2 mRNA-binding proteins (IGF2BP1/2/3; Huang et al., 2018; Imam et al., 2020) and other proteins are also been considered as readers.

N^6^-methyladenosine modification regulates various cellular physiological processes such as cell autophagy (Jin et al., 2018), cell differentiation and development (Frye et al., 2018), embryonic stem cells (ESCs) tumorigenicity (Sun et al., 2021), signal transduction (Chen et al., 2019), transcription and chromatin state (Wei and He, 2021) and immune response (Ma et al., 2021; Dong et al., 2022). Not only that, m^6^A modification has also been implicated in many pathological processes, such as human metabolic diseases (Zhang et al., 2021), cardiovascular diseases (Yankova et al., 2021; Zhou et al., 2021a), Alzheimer’s disease (AD; Shafik et al., 2021), cancer (Zhou et al., 2021b) and viral infections (Yu et al., 2021). The majority of current studies on m^6^A modification and viral infection are related to human pathogenic viruses, especially oncogenic viruses, and there have been many studies on the regulatory effects of m^6^A modification on viruses of the *Herpesviridae* family. For example, m^6^A modification double-regulates viral replication by regulating the ORF50 (RTA) of Kaposi′s sarcoma associated herpesvirus (KSHV; Sun et al., 1998). m6A modification positively regulates the replication of human cytomegalovirus (HCMV; Rubio et al., 2018) and herpes simplex virus 1 (HSV-1; Feng et al., 2021). Host m^6^A modification can be specifically regulated by Epstein-Barr virus (EBV) encoded oncoproteins to enhance viral oncogenicity (Lang et al., 2019), and EBV utilizes m^6^A to interfere with the interaction between virus and host, thereby achieving long-term latency (Zheng et al., 2021).

Here, we investigated the regulatory effect of m^6^A modification on PRV infection in porcine kidney epithelial cells (PK15) which is the most widely used in PRV isolation, propagation and basic research (Yang et al., 2017). We found that changing the m^6^A modification level of PK15 cells or the expression of m^6^A regulators affected viral replication. The m^6^A modification level of host cells and the expression levels of m^6^A regulators were affected after viral infection of PK15 cells, and m^6^A modification sites were also present in the virus transcripts.

## 2. Materials and methods

### 2.1. Cells, viruses, and plasmids

PK15 cells were obtained from the Swine Disease Research Center, College of Veterinary Medicine, Sichuan Agricultural University. PK15 cells were cultured in DMEM (HyClone, Thermo Fisher Scientific, Waltham, MA, United States), supplemented with 10% FBS (Gibco, Waltham, MA, United States) and 1% penicillin streptomycin (SANGON, Shanghai, China), and placed in a humidified incubator (5% CO_2_, 37°C).

The PRV isolate FJ01 (PRV-FJ01; Yi et al., 2019) was provided by Swine Disease Research Center, College of Veterinary Medicine, Sichuan Agricultural University.

Full-length PRV gB gene sequence was cloned into pMD19-T vector. GenBank accession: NC_006151.1. CDS-length porcine METTL14 cDNA was cloned into the pEGFP-C3 expression plasmid using the *Eco*RI and *Kpn*I restriction sites. GenBank accession: XM_003129231.6. The positive recombinant plasmids were sent for sequencing (SANGON).

### 2.2. Antibodies

Antibodies used in the study included rabbit anti-METTL14 (A8530, ABclonal, Wuhan, China), rabbit anti-ALKBH5 (DF2585, Affinity Biosciences, Jiangsu, China), rabbit anti-FTO (DF8421, Affinity Biosciences), Fluor647-conjugated goat anti-mouse IgG (S0014, Affinity Biosciences), rabbit anti-β-actin (AF7018, Affinity Biosciences), HRP-conjugated goat anti-rabbit IgG (31,460, Invitrogen, Waltham, MA, United States), HRP-conjugated goat anti-mouse IgG (D110087, SANGON), rabbit anti-YTHDF1 (17479-1-AP, Proteintech, Rosemont, IL, United States), rabbit anti-YTHDF2 (24744-1-AP, Proteintech), rabbit anti-YTHDF3 (25537-1-AP, Proteintech), and FITC AffiniPure goat anti-rabbit IgG (E031220-01, EarthOx, San Francisco, CA, United States). Mouse antiserum against PRV glycoprotein gE was kindly donated by Plecko bioengineering, Inc (Luoyang, China).

### 2.3. Quantification of RNA N^6^-methyladenosine modification level

PK15 cells were infected with PRV (MOI = 0.1), after 1 h adsorption, and cultured in DMEM (Hyclone) supplemented with 2% FBS (GIBCO) and 1% penicillin streptomycin (SANGON) at 37°C under 5% CO_2_ incubator. To quantify RNA m^6^A level, total RNA was extracted from infected and uninfected cells using the cell total RNA isolation kit (2.0–1902, FORGENE, Chengdu, China) at different time points. The purity and concentration of total RNA samples were determined with NanoDrop One Microvolume UV-Vis Spectrophotometer (Thermo Fisher Scientific). Quantification was performed using the EpiQuik m^6^A RNA methylation Quantification Kit (P-9005, Epigentek Group Inc., Farmingdale, NY, United States). Briefly, total RNA was bound to the strip wells using RNA high-binding solution. Specific m^6^A capture and detection antibodies were incubated with RNA bound in strip wells. Absorbance was read at 450 nm wavelength after detection of signal enhancement. The amount of m^6^A was proportional to the measured OD intensity. Three replicate samples were used to ensure credibility of the signal. A standard curve was established based on the provided standard positive samples, and the percentage of samples m^6^A was finally calculated.

### 2.4. RNA interference, transfection, and 3-Deazaadenosine treatment

siRNAs (60 nM) were transfected into PK15 cells using RNATransMate (E607402, SANGON) according to the manufacturer’s instructions. The siRNAs specifically targeting the m^6^A regulators were designed and synthesized by SANGON (Shanghai, China) to study viral replication. Two specific siRNAs were designed for METTL3, METTL14, FTO, ALKBH5, YTHDF1, YTHDF2 and YTHDF3 respectively, and non-specific siNC as a negative control was set (Supplementary Table S1). The knockdown efficacy was assessed by immunoblotting analysis and RT-qPCR at 48 h post-transfection. Similarly, PK15 cells (2 × 10^6^ cells) were transfected with pEGFP-C3-METTL14 (2.5 μg) using M5 HiPer Lipo2000 transfection reagent (MF135-01, Mei5bio, Beijing, China) to overexpress METTL14. METTL14 overexpression was assessed by immunoblotting analysis and RT-qPCR.

To inhibit the m^6^A modification level of PK15 cells, the original medium was replaced with the medium containing different concentrations of m^6^A modification inhibitor 3-Deazaadenosine (B6121, APExBIO, Houston, United States) after cell attachment. After cultured for 48 h, the inhibition level was assessed by epiquik m^6^A RNA methylation Quantification Kit (Epigentek Group Inc.).

### 2.5. MTT assay

MTT cell proliferation and cytotoxicity test kit (M1020, Solarbio, Beijing, China) was used to detect the effects of siRNAs, transfection reagent and 3-DAA on the viability of PK15 cells. PK15 cells were seeded at 1 × 10^4^ per well in 96-well plates. The medium containing various concentrations of 3-DAA or various siRNAs and transfection reagent was changed the next day. 10 μL MTT was added to each well after 48 h, and the culture was continued for 4 h. The medium was gently sucked out, and 110 μl formazan solution was added to each well, shaken and cultured for 10 min to completely dissolve the crystals. The absorbance value at 490 nm was measured by iMark microplate reader (Bio-Rad, Hercules, CA, United States) to determine the concentration of 3-DAA that had no effect on cell viability and whether siRNAs and transfection reagent had an effect on cell viability.

### 2.6. Virus infection and pseudorabies virus titration

PK15 cells were transfected with siRNAs against m^6^A regulators for 48 h, and then infected with PRV (MOI = 0.1). Similarly, the METTL14-overexpression cells were infected with PRV (MOI = 0.1). After infection for 1 h, fresh medium containing 2% FBS (Gibco) was added, and viral fluids were collected at different times. To detect the effect of 3-DAA on PRV titers, PK15 cells were treated with medium containing different concentrations of 3-DAA (ApexBio) for 24 h, and then infected with PRV (MOI = 0.1). After infection for 1 h, the medium containing 2% FBS (Gibco) and different concentrations of 3-DAA (ApexBio) were added, and viral fluids were collected at different times after infection. PK-15 cells were seeded at 1 × 10^4^ per well in 96-well plates, the collected viral fluids were used for PRV titration. The viral titers were determined by TCID_50_ according to Reed-Muench method.

### 2.7. Real-time quantitative PCR analysis

Total RNA was extracted using the cell total RNA Isolation Kit (FORGENE) and then reverse transcribed with the PrimeScript RT reagent Kit (RR047A, Takara, Shiga, Japan). RT-qPCR was performed in triplicate using SYBR premix Ex TaqII Kit (RR820, TaKaRa). The mRNA level was quantified by measuring the cycle threshold (CT) value. Data was normalized to the level of the control gene encoding GAPDH. The ΔΔCt method was used to measure the expression levels of target genes. According to the concentration of pMD19-T-gB and the corresponding CT value, the standard curve was established and the calculation formula of PRV copies was obtained. PRV DNA was extracted from different viral fluids by E.Z.N.A. Viral DNA Kit (Omega Bio-tek, Doraville, GA, United States). The PRV copies were detected by RT-qPCR using the extracted virus DNA. The primers are listed in Supplementary Table S2.

### 2.8. Immunoblotting analysis

PK15 cells were lysed on ice with lysis buffer (R0030, Solarbio) for 30 min. The protein concentration was measured with BCA protein quantitative measurement kit (PC0020, Solarbio), and then denatured by boiling in 5 × SDS loading buffer (P0015L, Beyotime, Shanghai, China) for 10 min, and separated by sodium dodecyl sulfate-polyacrylamide gel electrophoresis (SDS-PAGE). The protein was transferred to polyvinylidene difluoride (PVDF) membrane (JL19A03, Absin, Shanghai, China). After blocking at room temperature in PBST containing 5% skimmed milk powder (A600669, SANGON) for 1.5 h, the membrane was incubated with different primary antibodies at 4°C overnight, added with the corresponding secondary antibodies containing HRP and incubated at room temperature for 2 h. The blot was observed by BeyoECL Star (P0018A, Beyotime).

### 2.9. Methylated RNA immunoprecipitation sequencing and data analysis

PRV (MOI = 1) infected PK15 cells for 24 h, and uninfected cells were used as negative control. Total RNA was extracted using the cell total RNA Isolation Kit (FORGENE). The purity and concentration of total RNA samples were determined with NanoDrop ND-1000 (Thermo Fisher Scientific). According to the manufacturer′s protocol, the intact mRNA in the sample was captured with Seq-Star™ poly(A) mRNA Isolation Kit (AS-MB-006-01/02, ArrayStar, Rockville, MD, United States), and then the isolated mRNA was chemically fragmented into 300-nucleotide-long fragments by incubation in the fragmentation buffer (10 mM Zn^2+^ and 10 mM Tris-HCl, pH 7.0). Fragmented mRNA was immunoprecipitated with affinity purified anti-m^6^A rabbit antibody (202,003, Synaptic Systems, Göttingen, Germanys), and one-tenth of the fragment mRNA was retained as input. After immunoprecipitation, washing and elution, m^6^A-modified mRNA and input mRNA were enriched respectively, and RNA-seq libraries were constructed by KAPA Stranded mRNA-seq Kit (Illumina; KK8421, KAPA Biosystems, Boston, Massachusetts, United States). The completed libraries were qualified by the Agilent 2,100 Bioanalyzer Bioanalyzer (Agilent Technologies, Santa Clara, CA, United States). The sequencing libraries was denatured in 0.1 M NaOH to generate single-stranded DNA. DNA clusters were generated on Illumina cBot with the NovaSeq 6,000 S4 Reagent Kit (20,012,866, Illumina Inc., San Diego, CA, United States). Finally, the clustering libraries were loaded onto reagent cartridge and forwarded to sequencing run on the NovaSeq 6,000 system (Illumina Inc.). The raw sequencing data was quality-analyzed by FastQC (v0.11.5). After removing the low-quality sequences using Trimmomatic (v0.32), the filtered sequences were compared with the PRV reference genome (GenBank accession: NC_006151.1) using HISAT2 (v2.1.0), and then the m^6^A peaks was analyzed using exomePeak (v2.13.2; Meng et al., 2014). According to the peaks obtained from the annotation information of the database, whether there were peaks in each sample in each transcriptional region was calculated. The DREME method in MEME-ChIP (v4.12.0) was used to detect the motif, and finally analyzed the obtained peaks. The original sequencing data obtained from methylated RNA immunoprecipitation sequencing (MeRIP-seq) reported in this study have been deposited in NCBI GEO under accession number GSE209949.

### 2.10. Statistical analysis

In the Immunoblotting analysis, the corresponding immunoreactive protein band intensities were determined using Image J (v1.53). All statistical analyses were performed using the two-tailed Student’s t-test with GraphPad Prism 9 (GraphPad Software, San Diego, CA, United States). *p* < 0.05 was considered statistically significant. All data were obtained from three-time experiments independently for quantitative analyses.

## 3. Results

### 3.1. N^6^-methyladenosine modifications are widespread in pseudorabies virus transcripts in PK15 cells

**Figure 1:**
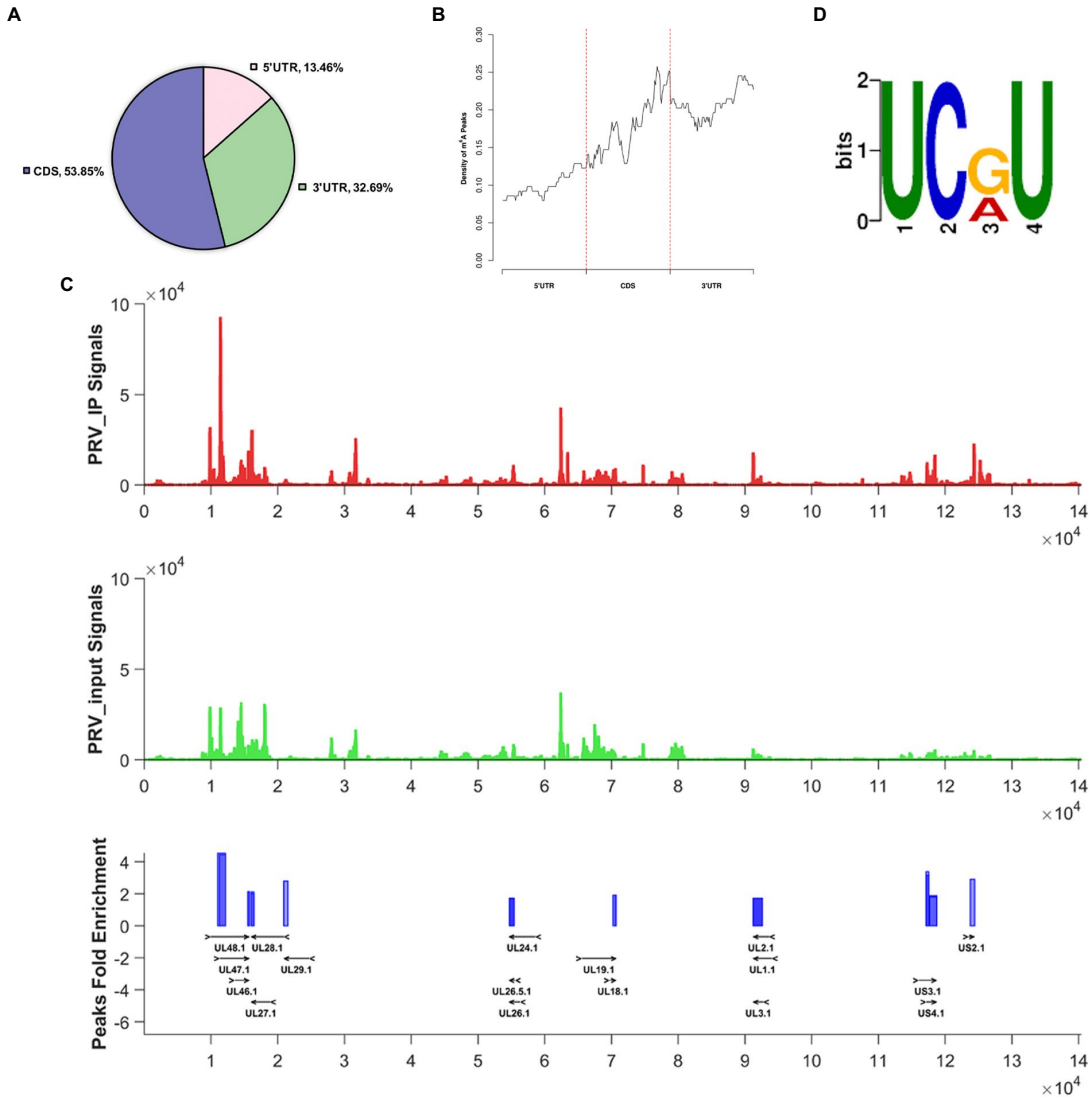
The status of m^6^A modification on PRV transcripts. For PRV (MOI = 1) infection, total RNA of PK15 cells was harvested at 24 hpi. **(A)** Distribution pattern of m^6^A peaks on PRV transcripts was analyzed based on the MeRIP-seq data (NCBI #GSE209949). **(B)** Density of m^6^A peaks on PRV transcripts. **(C)** Transcriptome-wide mapping to PRV m^6^A IP reads, input reads and m^6^A peaks based on MeRIP-seq. The m^6^A peaks of PRV transcripts were indicated as blue blocks. The input and PRV IP coverage were indicated with green and red bars, respectively. All genes were shown and overlaid as black arrows in the bottom track. **(D)** Motif analysis to identify consensus sequences for PRV transcripts. The most prominent motif was shown.

To identify possible m^6^A modification sites in the PRV epitranscriptome, we performed MeRIP-seq (Dominissini et al., 2013) of total RNA isolated from PRV-infected PK15 cells to understand m^6^A modification sites in host mRNA and viral mRNA. To better understand post-transcriptional modifications, especially m^6^A modification sites on viral transcripts, we compared MeRIP-seq data with the PRV reference sequence (GenBank accession: NC_006151.1) and showed that PRV transcripts containing m^6^A modifications widely existed in PRV-infected PK15 cells. A total of 21 m^6^A peaks were found in the PRV transcripts (Table 1). These m^6^A peaks were mainly distributed in the CDS region (Figure 1A), especially the 3′ end had the highest content, and the 3′ UTR region was also widespread (Figure 1B). The multiple m^6^A methylated viral transcripts included were UL1, UL2, UL3, UL18, UL19, UL24, UL26, UL26.5, UL27, UL28, UL29, UL46, UL47, UL48, US2, US3, and US4 (Figure 1C). The most significant motif in the m6A peaks of the PRV transcripts was identified as UCRU (U-C-G/A-U; E-value was 5.9e-005) using the DREME method in MEME-ChIP (Figure 1D).

**Table 1:** Integration statistics of significant m^6^A peaks information.

Peak start	Peak end	Gene name	Strand	*P* value	Fold change
91,212	92,670	UL1	−	0.0331131	1.72548
91,212	92,668	UL2	−	0.0331131	1.72668
91,212	92,668	UL3	−	0.0331131	1.72668
70,221	70,728	UL18	+	0.00891251	1.92252
70,219	70,728	UL19	+	0.00891251	1.92119
54,667	55,442	UL24	−	0.042658	1.71119
54,667	55,438	UL26	−	0.0380189	1.73267
54,666	55,424	UL26.5	−	0.0416869	1.71475
16,000	16,448	UL27	−	0.00186209	2.01391
16,000	16,419	UL28	−	0.001	2.12874
20,897	21,557	UL29	−	0.00933254	2.78949
15,519	15,759	UL46	+	0.0275423	2.14355
15,519	15,759	UL47	+	0.0275423	2.14355
11,277	12,204		+	5.01187E-40	4.43828
15,518	15,759	UL48	+	0.0275423	2.14355
11,023	12,193		+	3.16228E-43	4.53154
123,755	124,429	US2	+	0.000776247	2.90795
117,678	118,755	US3	+	0.00107152	1.87774
117,200	117,590		+	0.00011749	3.16017
117,128	117,572	US4	+	3.80189E-05	3.38698
117,690	118,755		+	0.0017378	1.80876

Peak start: position from which the peak starts. Peak end: position with which the peak ends. Strand: the strand direction of the peak on scaffold. *P* value: statistical significance for the differential methylation. Fold change: fold change between PRV_IP and PRV_input.

### 3.2. Pseudorabies virus infection influences N^6^-methyladenosine methylation pattern of PK15 cells

**Figure 2:**
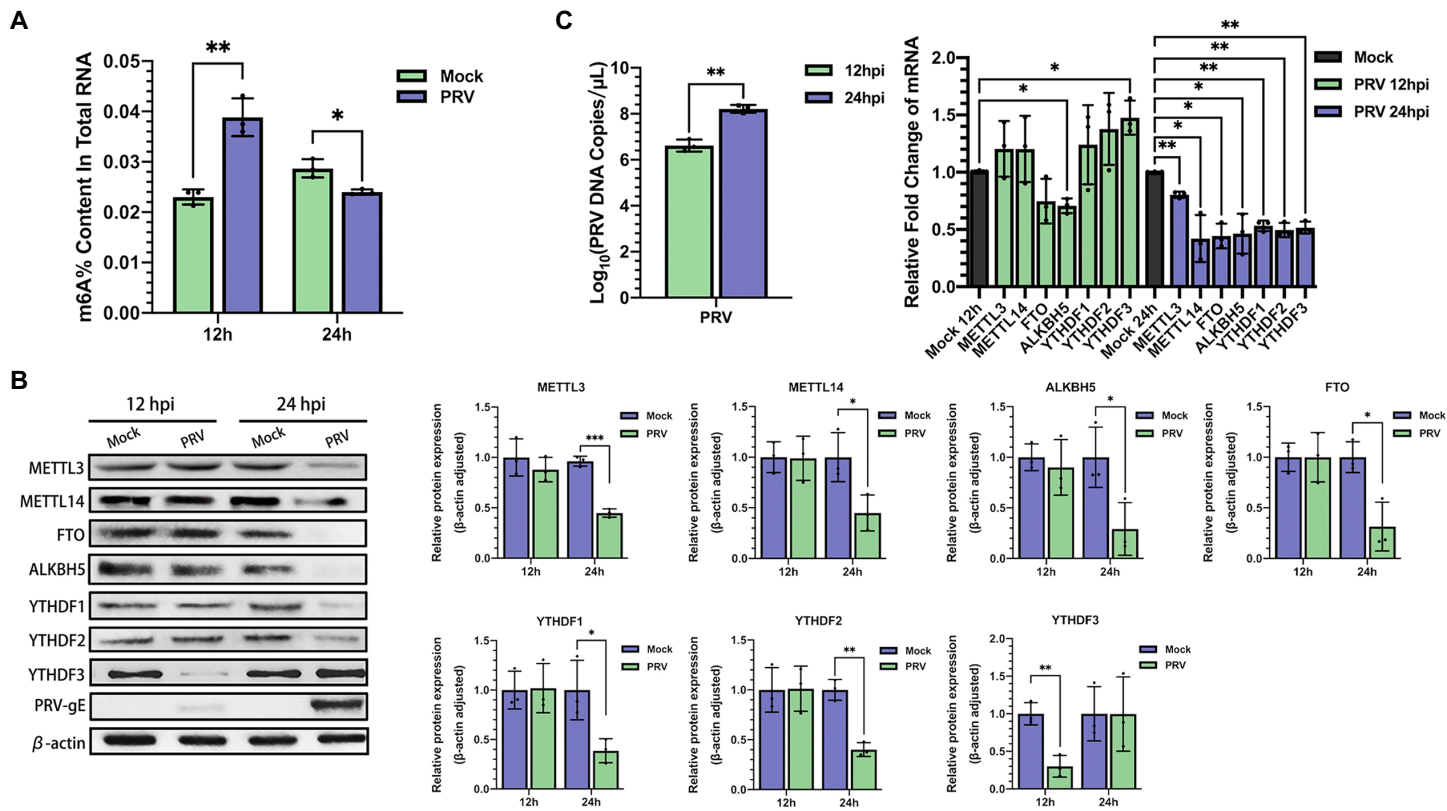
PRV infection affected m^6^A level and expression of m^6^A regulators in PK15 cells. **(A)** Total RNA was extracted from PRV-infected and uninfected PK15 cells at different time periods, and the m^6^A level of RNA was quantified by ELISA. **(B)** PK15 cells were infected with PRV for 12 and 24 h. m^6^A regulators were assessed by immunoblotting analysis. β-actin was used as a loading control. **(C)** RT-qPCR analysis was used to evaluate the mRNA levels of m^6^A regulators at different times of PRV infection. *p < 0.05, **p < 0.01, ***p < 0.001.

N^6^-methyladenosine modification is present in viral and host RNAs. Now that we have shown that m^6^A peaks exist in PRV transcripts, we next investigated its effect on host RNA m^6^A modification. Total RNA and protein were harvested at different time points after PK15 cells were infected with PRV. Using the m^6^A RNA methylation quantification Kit, we first examined the m^6^A ratio in total RNA. PRV infection could lead to an increased in m^6^A ratio in PK15 cells at 12 hpi compared with uninfected, indicating that PRV infection enhanced host m^6^A RNA methylation, but the m^6^A ratio decreased after 24 hpi (Figure 2A). This might be due to the fact that PRV infection destroyed most cells as the viral replication process progressed. Then, we detected the expression patterns of m^6^A methyltransferases, demethylases, and specific binding proteins in PK15 cells. The immunoblotting analysis showed that the protein expression of all m^6^A regulators except YTHDF3 decreased at 24 hpi, while remained stable at 12 hpi. The protein expression of YTHDF3 began to decrease at 12 hpi, but was stable at 24 hpi (Figure 2B). The protein expression levels were correlated with the mRNA levels of the encoded protein. RT-qPCR was used to detect the mRNA levels of m^6^A regulators. The mRNA levels of ALKBH5 decreased and YTHDF3 increased after 12 hpi, while the mRNA levels of other proteins did not change significantly. The mRNA levels of all m^6^A regulators were significantly decreased after 24 hpi (Figure 2C), which illustrated that PRV infection reduced the mRNA abundance of m^6^A regulators. The inconsistency between YTHDF3 mRNA and protein expression levels indicated that PRV infection might induce changes in protein expression level through other pathways besides affecting mRNA abundance, such as protein post-translational modification or altering protein efficiency, which specifically needed to be further investigated.

**Figure 3:**
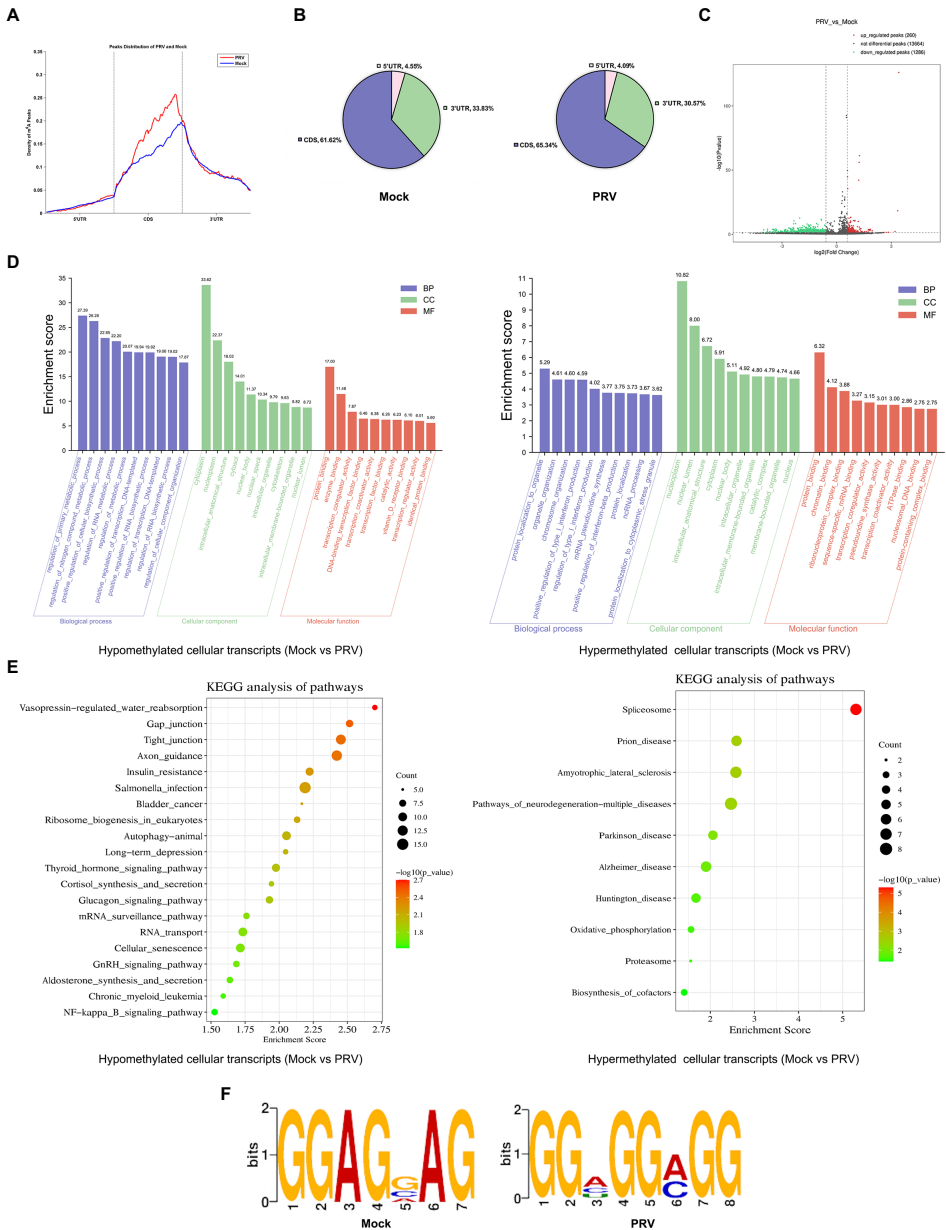
PRV infection influenced m^6^A methylome of PK15 cell transcripts. **(A)** MeRIP-seq of PK15 cells which were infected by PRV (or uninfected as a negative control, i.e., “Mock”) for 24 h. Density of m^6^A peaks on PRV-infected and uninfected cellular transcripts. The m^6^A peaks information was included in our MeRIP-seq data (NCBI #GSE209949). **(B)** Distribution pattern of m^6^A peaks on PRV-infected (right) and uninfected (left) cellular transcripts. **(C)** Volcanic map of m^6^A peaks (left was downregulated, right was upregulated by PRV infection). There were 1,286 significantly down-regulated m^6^A peaks, and 260 significantly up-regulated m^6^A peaks induced by PRV infection. **(D)** GO enrichment analysis of pathways enriched in the hypomethylated (left) and hypermethylated (right) genes (The top 30 enriched pathways are shown.). **(E)** KEGG analysis of pathways enriched in the hypomethylated genes (left, the top 20 enriched pathways are shown.) and the hypermethylated genes (right, the top 10 enriched pathways are shown.). **(F)** Motif analysis to identify consensus sequences for PRV-infected (right) and uninfected (left) PK15 cells transcripts. The most prominent motif for each was shown.

To determine whether host m^6^A modification was affected by PRV infection, we performed MeRIP-seq on total RNA collected from PRV-infected PK15 cells, m^6^A peaks were detected using exomePeak, and the resulting m^6^A peaks were annotated according to the annotation information of the Ensembl database. Most m^6^A modification peaks were distributed in the 3′ UTR and CDS regions of PRV-infected and uninfected PK15 cells (Figure 3A). During viral infection, the distribution of m^6^A peaks on host transcripts did not change significantly. PRV infection increased the distribution of m^6^A peaks in the coding regions and decreased their distribution in the untranslated regions. In Mock group, 61.62% of m^6^A peaks were located in CDS, whereas in PRV-infected cells, the percentage rose to 65.34% (Figure 3B). The differential m^6^A peaks were screened by the difference significance and the difference multiples after normalization, and the volcano map was drawn. The red dots indicated the significant peaks that were up-regulated after PRV infection, the green dots indicated the significant peaks that were down-regulated after PRV infection. The gray dots indicated that the difference was not significant or the difference multiples were lower than the set threshold, in which the difference significance threshold was *p* < 0.05. Compared with 1,286 significantly down-regulated m^6^A peaks, PRV infection induced 260 significantly up-regulated m^6^A peaks (Figure 3C). Gene Ontology (GO) enrichment analysis was performed on the differential m^6^A peaks, and the results were described in combination with GO annotations. The top 10 significant enriched pathways in each process were plotted (Figure 3D). The most significant regulation of the hypomethylated genes in biological process was the regulation of primary metabolic process, followed by the positive regulation of cellular biosynthetic process, the regulation of RNA metabolic process and the positive regulation of transcription, etc. The most significant regulation in molecular function was protein binding, followed by enzyme binding, transcription coregulator activity and transcription factor binding, and etc. The most significant regulation in cellular composition was the cytoplasm. The most significant regulation of the hypermethylated genes in biological process was protein localization to organelles, followed by organelle organization and positive regulation of type I interferon production, etc. The most significant regulation of the molecular function was also protein binding, followed by sequence-specific mRNA binding and transcriptional coregulator activity, etc. The most significant regulation of the cellular component was nucleoplasm. Subsequently, Kyoto Encyclopedia of genes and genomes (KEGG) enrichment analysis was performed, and the bubble plot were made for the metabolic pathways of the top 20 differentially hypomethylated genes (Figure 3E). Differentially hypomethylated genes were mainly enriched in the vasopressin-regulated water reabsorption, gap junction, tight junction, axon guidance, insulin resistance, NF-κB signaling and other pathways. Bubble plots were made for the metabolic pathways of 10 differentially hypermethylated genes revealed that the differentially hypermethylated genes were mainly enriched in the spliceosome, prion disease, amyotrophic lateral sclerosis, pathways of neurodegeneration-multiple diseases, Alzheimer’s disease, oxidative phosphorylation, and other pathways (Figure 3E). The DREME method in MEME-ChIP was used to detect the most significant motif of m^6^A peaks obtained from PRV-infected and uninfected PK15 cells to explore whether the motif changed after viral infection. The most significant motif in PRV-uninfected PK15 cells was GGAGVAG (G-G-A-G-G/C/A-A-G; E-value was 6.9e-013), and the most significant motif in PRV-infected PK15 cells was GGHGGMGG (G-G-A/C/U-G-G-A/C-G-G; E-value is 1.1e-018; Figure 3F).

### 3.3. N^6^-methyladenosine writers promote pseudorabies virus replication

**Figure 4:**
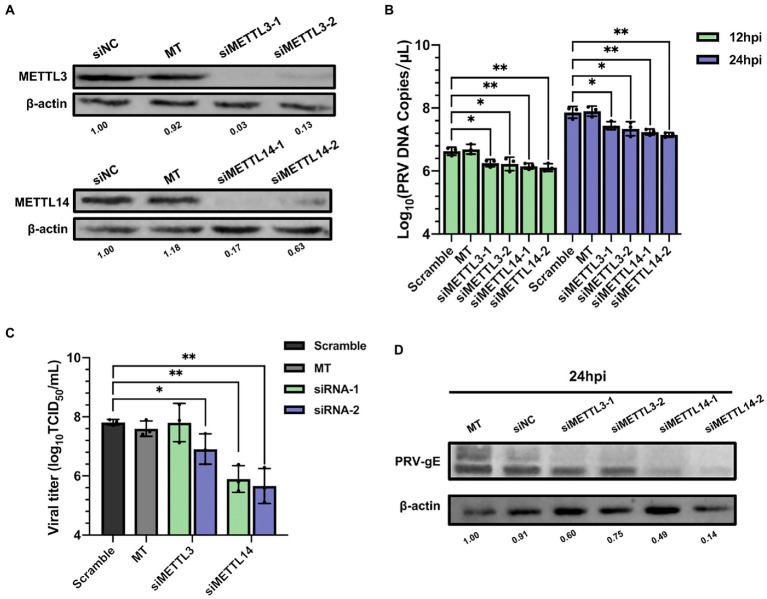
Depletion of methyltransferases METTL3 and METTL14 suppressed PRV replication. **(A)** PK15 cells were transfected with the specified siRNAs (60 nM) for 24 h. METTL3 and METTL14 were assessed by immunoblotting analysis. β-actin was used as a loading control. **(B)** PK15 cells were transfected with the specified siRNAs and were mock transfected (MT) with transfection reagent alone for 24 h. PK15 cells were infected with PRV-FJ01 (MOI = 0.1) for 12 and 24 h. PRV DNA copies were evaluated by RT-qPCR analysis. **(C)** PK15 cells were transfected with the specified siRNAs and were mock transfected (MT) with transfection reagent alone for 24 h. PK15 cells were infected with PRV-FJ01 (MOI = 0.1) for 24 h. PRV titers were assessed by TCID_50_ analysis. **(D)** PK15 cells were transfected with the indicated siRNAs and were mock transfected (MT) with transfection reagent alone for 24 h. PK15 cells were infected with PRV-FJ01 (MOI = 0.1) for 24 h. PRV gE was assessed by immunoblotting analysis. β-actin was used as a loading control. *p < 0.05, **p < 0.01, ***p < 0.001.

N^6^-methyladenosine modification is a dynamic and reversible process, which is catalyzed by m^6^A writers (Liu et al., 2014). We next examined the role of the m^6^A writers in PRV replication and protein expression. We used RNAi method to knockdown the corresponding genes, synthesized siRNAs targeting porcine METTL3 and METTL14, and transfected them into PK15 cells (60 nM). At 48 h after transfection, cellular proteins were collected for immunoblotting analysis, and total RNA was extracted for RT-qPCR analysis after reverse transcription. METTL3 and METTL14 mRNA levels decreased by more than half (Supplementary Figure S3), and the protein expression also decreased significantly (Figure 4A). Moreover, since the proliferation and viability of host cells affect the replication and proliferation of virus, it is necessary to understand whether the siRNAs of m^6^A regulators affect the cell activity. Therefore, we used MTT assay to detect the cell activity after METTL3 and METTL14 knockdown. Although METTL3 and METTL14 could inhibit the proliferation of tumor cells (Cui et al., 2017; Ma et al., 2017), we did not observe the effect of METTL3 and METTL14 knockdown on cell proliferation (Supplementary Figure S1). At 24 h after transfection, PRV (MOI = 0.1) infection was carried out. The viral fluids were collected at 12 and 24 hpi for virus titration and RT-qPCR analysis. The copies of viral DNA were determined by RT-qPCR analysis. After knockdown of METTL3 or METTL14, the copies of PRV decreased significantly, and the effect of METTL14 was more obvious (Figure 4B). In order to further verify whether this effect would reduce the viral titers, the results of TCID_50_ test showed that the viral titers decreased about 100 times after METTL14 knockdown compared with the control group transfected with siNC. Compared with METTL14, siMETTL3-1 for METTL3 had no effect on viral titers, while siMETTL3-2 reduced viral titers by about 10 times (Figure 4C). In addition, after silencing METTL3 or METTL14, the gE protein synthesized by the virus was also significantly reduced in PK15 cells (Figure 4D). These results suggested that m6A writers contribute to PRV replication and gene expression.

**Figure 5:**
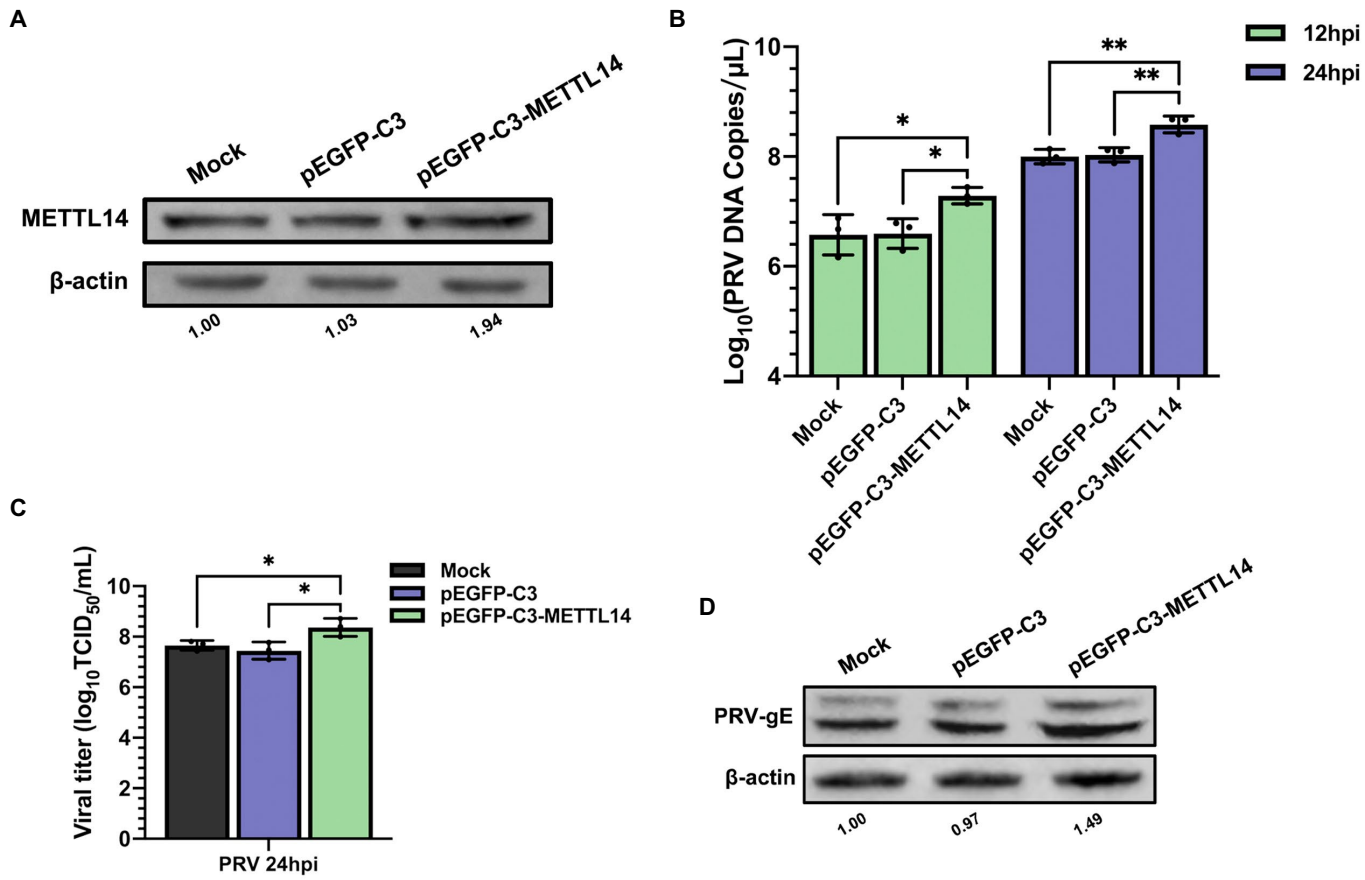
Overexpression of METTL14 promoted PRV proliferation. **(A)** PK15 cells were transfected with pEGFP-C3 and pEGFP-C3-METTL14 (2.5 μg) for 6 h, and then cultured with fresh maintenance medium for 24 h. METTL14 was assessed by immunoblotting analysis. β-actin was used as a loading control. **(B)** PK15 cells were transfected with pEGFP-C3 and pEGFP-C3-METTL14 (2.5 μg) for 6 h, and then cultured with fresh maintenance medium for 24 h. PK15 cells were infected with PRV-FJ01 (MOI = 0.1) for 12 and 24 h. PRV DNA copies were evaluated by RT-qPCR analysis. **(C)** PK15 cells were transfected with pEGFP-C3 and pEGFP-C3-METTL14 (2.5 μg) for 6 h, and then cultured with fresh maintenance medium for 24 h. PK15 cells were infected with PRV-FJ01 (MOI = 0.1) for 24 h. PRV titers were assessed by TCID_50_ analysis. **(D)** PK15 cells were transfected with pEGFP-C3 and pEGFP-C3-METTL14 (2.5 μg) for 6 h, and then cultured with fresh maintenance medium for 24 h. PK15 cells were infected with PRV-FJ01 (MOI = 0.1) for 24 h. PRV gE was assessed by immunoblotting analysis. β-Actin was used as a loading control. *p < 0.05, **p < 0.01.

Since depletion of METTL14 suppressed PRV replication, we further probed whether METTL14 overexpression affected PRV replication. The mRNA level of METTL14 increased significantly after transfection of pEGFP-C3-METTL14 (Supplementary Figure S4), and there was no effect on cell activity (Supplementary Figure S2). Furthermore, we used immunoblotting analysis to further examine the protein expression of METTL14 after transfection with pEGFP-C3-METTL14, and the protein expression of METTL14 also increased after transfection (Figure 5A). The PRV copies (Figure 5B), titers (Figure 5C), and the expression of gE protein (Figure 5D) were significantly increased after METTL14 overexpression. This was in contrast to the situation with METTL14 knockdown.

### 3.4. Depletion of N^6^-methyladenosine erasers enhance pseudorabies virus replication

**Figure 6:**
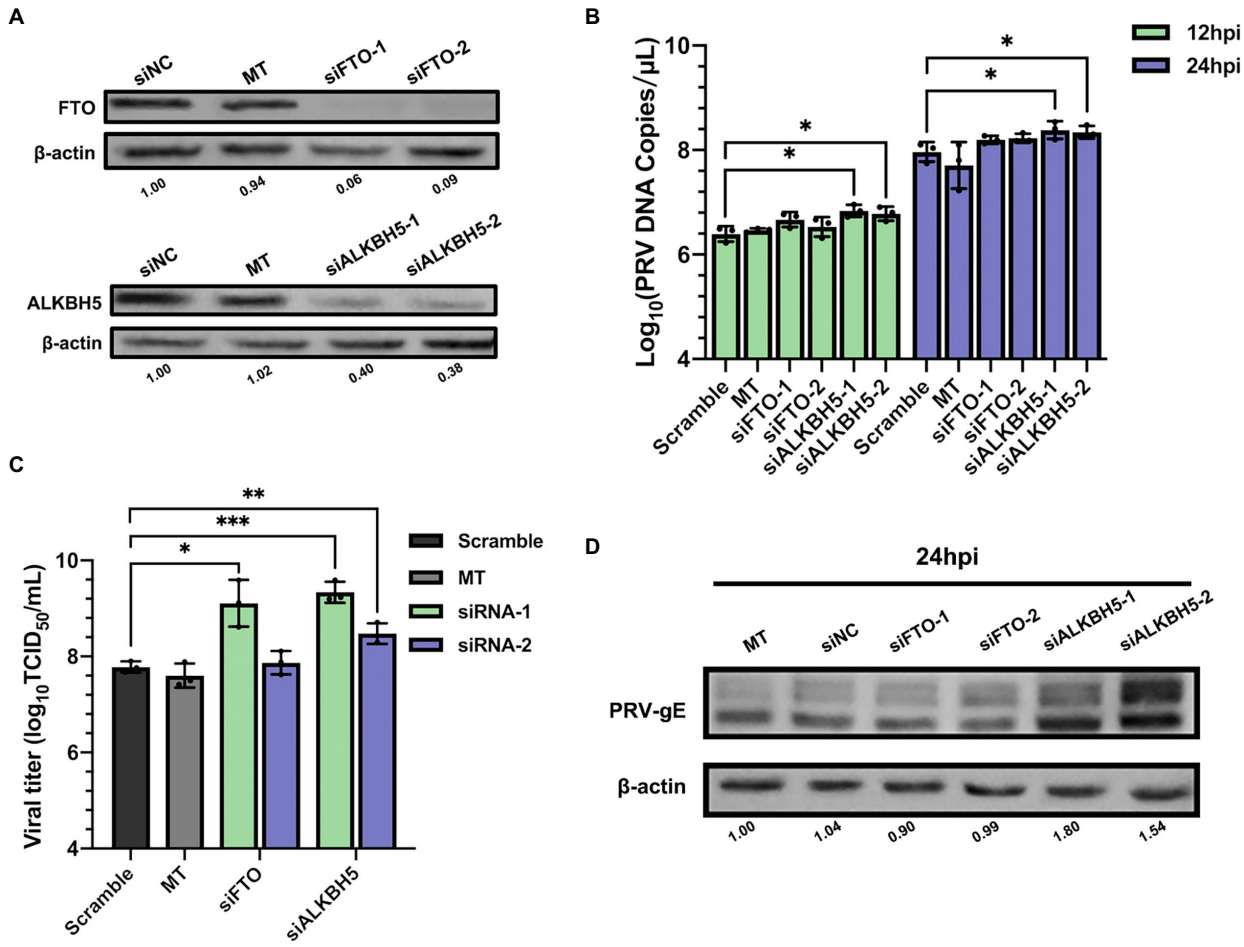
Demethylase FTO and ALKBH5 promoted PRV proliferation. **(A)** PK15 cells were transfected with the specified siRNAs (60 nM) for 24 h. FTO and ALKBH5 were assessed by immunoblotting analysis. β-actin was used as a loading control. **(B)** PK15 cells were transfected with the specified siRNAs and were mock transfected (MT) with transfection reagent alone for 24 h. PK15 cells were infected with PRV-FJ01 (MOI = 0.1) for 12 and 24 h. PRV DNA copies were evaluated by RT-qPCR analysis. **(C)** PK15 cells were transfected with the indicated siRNAs and were mock transfected (MT) with transfection reagent alone for 24 h. PK15 cells were infected with PRV-FJ01 (MOI = 0.1) for 24 h. PRV titers were assessed by TCID_50_ analysis. **(D)** PK15 cells were transfected with the indicated siRNAs and were mock transfected (MT) with transfection reagent alone for 24 h. PK15 cells were infected with PRV-FJ01 (MOI = 0.1) for 24 h. PRV gE was assessed by immunoblotting analysis. β-actin was used as a loading control. *p < 0.05, **p < 0.01, ***p < 0.001.

N^6^-methyladenosine modification is reversible and can be removed by m^6^A erasers (Jia et al., 2011; Zheng et al., 2013). Therefore, we studied the effect of erasers FTO or ALKBH5 knockdown on PRV replication. We performed loss of function knockdown test on FTO and ALKBH5 by specific siRNAs. As shown in Supplementary Figure S3, knockdown efficiency was greater than 40%, protein expression was also significantly reduced (Figure 6A). siRNAs targeting FTO and ALKBH5 did not change cell survival (Supplementary Figure S1). Compared with siNC transfected cells, knockdown of ALKBH5 significantly increased the PRV copies, but knockdown of FTO had no effect (Figure 6B). For TCID_50_ detection, using siFTO-1 could increase PRV titers, but siFTO-2 had no effect. This might be due to the higher knockdown efficiency of siFTO-1. Knockdown of ALKBH5 could increase PRV titers by about 1.25 times (Figure 6C). In addition, the expression of PRV gE protein increased by more than 2 times (Figure 6D). Therefore, knockdown of m^6^A erasers had a positive effect on PRV replication and gene expression.

### 3.5. Depletion of N^6^-methyladenosine readers suppress pseudorabies virus replication

**Figure 7:**
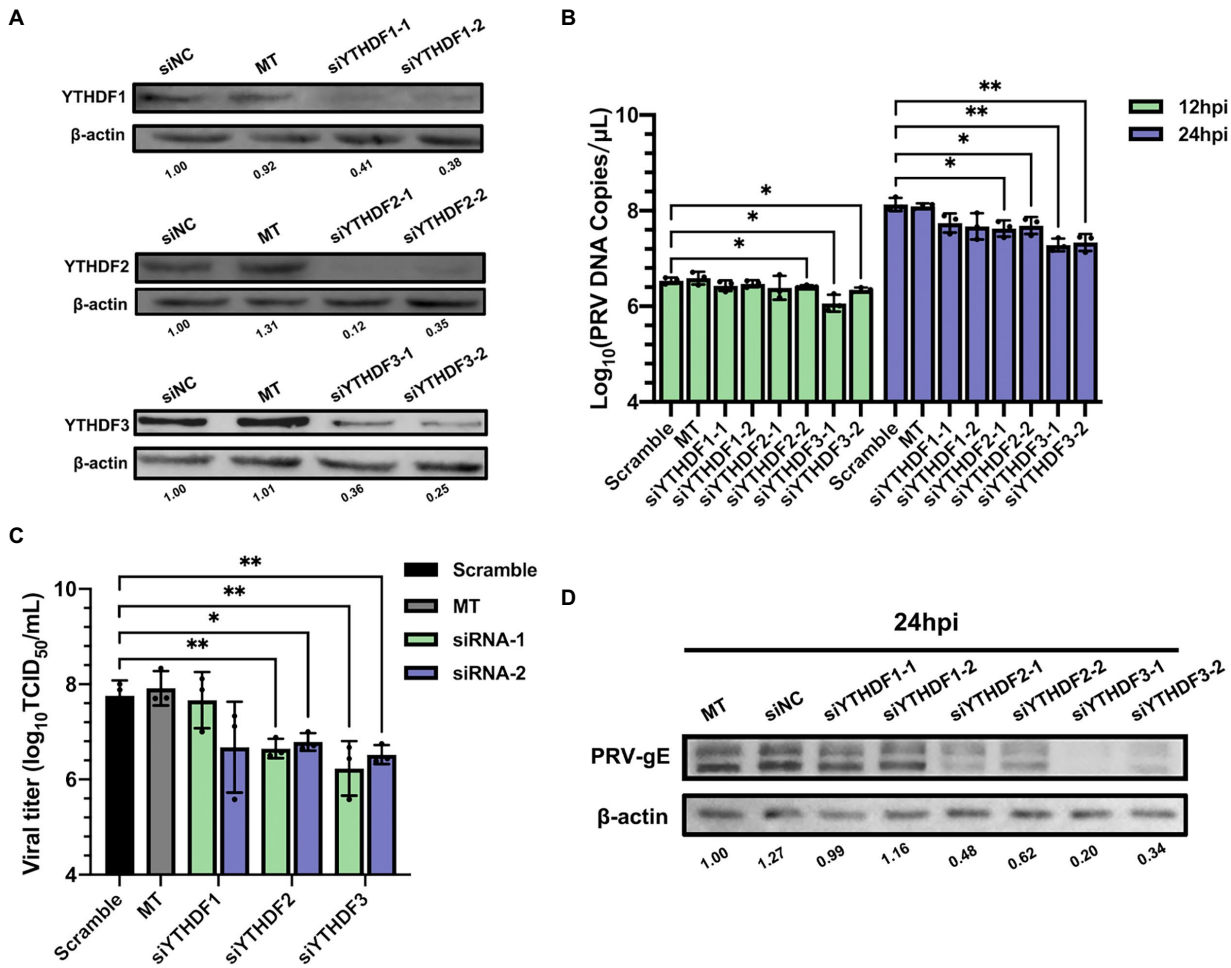
Specific recognition protein YTHDF2 and YTHDF3 inhibited PRV proliferation. **(A)** PK15 cells were transfected with the specified siRNAs (60 nM) for 24 h. YTHDF1, YTHDF2 and YTHDF3 were assessed by immunoblotting analysis. β-actin was used as a loading control. **(B)** PK15 cells were transfected with the specified siRNAs and were mock transfected (MT) with transfection reagent alone for 24 h. PK15 cells were infected with PRV-FJ01 (MOI = 0.1) for 12 and 24 h. PRV DNA copies were evaluated by RT-qPCR analysis. **(C)** PK15 cells were transfected with the indicated siRNAs and were mock transfected (MT) with transfection reagent alone for 24 h. PK15 cells were infected with PRV-FJ01 (MOI = 0.1) for 24 h. PRV titers were assessed by TCID_50_ analysis. **(D)** PK15 cells were transfected with the specified siRNAs and were mock transfected (MT) with transfection reagent alone for 24 h. PK15 cells were infected with PRV-FJ01 (MOI = 0.1) for 24 h. PRV gE was assessed by immunoblotting analysis. β-actin was used as a loading control. *p < 0.05, **p < 0.01.

N^6^-methyladenosine readers can bind m^6^A containing motifs. Since both m^6^A writers and erasers were involved in regulating PRV infection, we next tested whether m^6^A readers YTHDF1-3 could regulate virus infection. For each m^6^A reader, we applied two different siRNAs to knockdown specific genes in PK15 cells. Except siYTHDF3-2, the knockdown efficiency exceeded 50% (Supplementary Figure S3), the expression of corresponding proteins decreased significantly (Figure 7A), and had no effect on cell activity (Supplementary Figure S1). In addition to YTHDF1, knockdown of YTHDF2 or YTHDF3 could significantly reduce the viral copies (Figure 7B) and titers (Figure 7C). Knockdown of YTHDF3 was more obvious and reduced the production of virus particles by more than 10 times. After knockdown of YTHDF2 or YTHDF3, the expression level of viral gE protein in PK15 cells decreased significantly, especially when knockdown of YTHDF3 (Figure 7D). Therefore, m^6^A specific binding proteins promoted virus replication.

### 3.6. N^6^-methyladenosine methylation inhibitor suppresses pseudorabies virus replication

**Figure 8:**
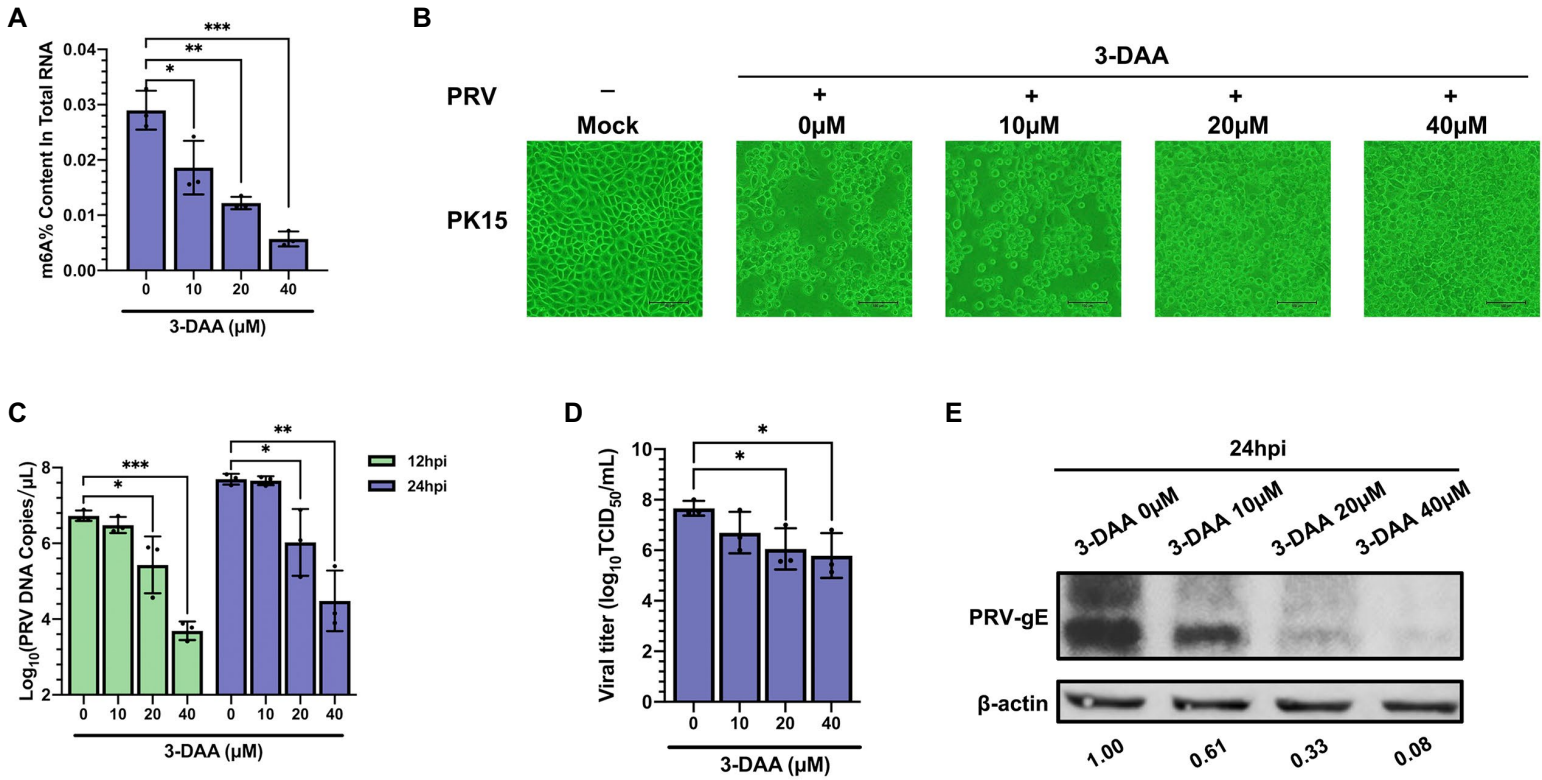
Inhibition of PRV infection by methylation inhibitor 3-deazaadenosine (3-DAA). **(A)** PK15 cells were treated with the specified concentrations of 3-DAA for 24 h. m^6^A level quantification was performed by ELISA assays. **(B)** PK15 cells were treated with the specified concentrations of 3-DAA for 24 h. PK15 cells were infected with PRV-FJ01 (MOI = 0.01) for 24 h, and images of cytopathic effects were recorded (200×). **(C)** PK15 cells were treated with the specified concentrations of 3-DAA for 24 h. PK15 cells were infected with PRV-FJ01 (MOI = 0.01) for 24 h. PRV DNA copies were evaluated by RT-qPCR analysis. **(D)** PK15 cells were treated with the specified concentrations of 3-DAA for 24 h. PK15 cells were infected with PRV-FJ01 (MOI = 0.01) for 24 h. PRV titers were assessed by TCID_50_ analysis. **(E)** PK15 cells were treated with the specified concentrations of 3-DAA for 24 h. PK15 cells were infected with PRV-FJ01 (MOI = 0.01) for 24 h. PRV gE was assessed by immunoblotting analysis. β-actin was used as a loading control. *p < 0.05, **p < 0.01, ***p < 0.001.

3-deazaadenosine (3-DAA) inhibits the hydrolysis of S-adenosylhomocysteine (SAH) to form S-adenosylmethionine (SAM) to block the m^6^A catalytic reaction of RNA, which is a comprehensive inhibitor of m^6^A methylation (Duerre et al., 1992). We used 3-DAA as an inhibitor of m^6^A modification to establish a PK15 cell model with m^6^A modification level inhibition. The m^6^A modification level of total RNA in the 3-DAA treatment group gradually decreased with the increase of 3-DAA concentration (Figure 8A). We chose the concentration of 3-DAA that had no effect on cell activity (Supplementary Figure S5). 3-DAA protected PK15 cells from PRV infection in a dose-dependent manner, weakening the cytopathic effect (CPE; Figure 8B). 3-DAA treatment reduced the viral replication (Figure 8C) and the titers of viral particles (Figure 8D). The expression level of PRV gE protein also gradually decreased with the increase of 3-DAA concentration (Figure 8E). These results emphasized that the deletion of m^6^A modification in the virus infection cycle inhibited viral replication and reproduction.

## 4. Discussion

N^6^-methyladenosine modification exists in RNA of virus and host. m^6^A modification is known to participate in the game of diverse viruses and host cells, a detailed understanding of RNA epigenetics will facilitate the development of antiviral drugs. We found that m^6^A modification was widespread in PRV transcripts of infected cells (Figure 1). The proteins encoded by the m^6^A peaks of the PRV transcripts were involved in nearly every progression of the PRV life cycle, such as viral entry and intercellular diffusion (Pomeranz et al., 2005), DNA replication, repair and recombination (Ben-Porat et al., 1983), assembly of viral particles (Kopp et al., 2002), viral nuclear export (Wagenaar et al., 1995), capsid assembly and maturation (Pomeranz et al., 2005), among others. Similar to EBV (Xia et al., 2021; Yanagi et al., 2022) and KSHV (Baquero-Perez et al., 2019), which belonged to the *Herpesviridae* family, the majority of m^6^A peaks were distributed on the CDS of viral mRNAs, especially the 3′ end, suggesting that m^6^A modification might regulate viral gene transcription or translation and play a role in viral assembly and replication.

Furthermore, PRV infection triggered reprogramming of m^6^A methylome in host cells RNA (Figures 2, 3). Analysis of the distribution pattern of m^6^A peaks revealed that cellular m^6^A peaks were mainly distributed in both 3′ UTR and CDS regions, which was similar to the topological pattern of m^6^A methylation in porcine liver transcriptome (He et al., 2017). PRV infection resulted in an increased content of m^6^A peaks in CDS regions and a concomitant decrease in m^6^A peaks in UTRs, which might represent a cellular response to the stress of viral infection. The significantly up-regulated m^6^A peaks induced by PRV infection were much lower than the significantly down-regulated m^6^A peaks. To further understand the role of these differential m^6^A transcripts in PK15 cells after PRV infection, GO enrichment and KEGG analysis were used to analyze the function of these differential m^6^A-modified mRNAs. GO enrichment analysis showed that hypomethylated genes were mainly involved in the metabolic process, indicating that m^6^A modification might be involved in the process of metabolic dysfunction related to PRV infection. Hypermethylated genes were mainly involved in protein localization, protein binding and type I interferon production. m^6^A modification was involved in regulating the host immune response to various viral infections (Mcfadden and Horner, 2021), and many viruses utilized m^6^A modification to evade host innate immunity (Kim et al., 2020; Lu et al., 2020, 2021; Xue et al., 2021). PRV had evolved a variety of host immune escape mechanisms (Ye et al., 2022), so m^6^A modification might be involved in regulating host immune escape after PRV infection. KEGG analysis showed that the water reabsorption pathway was the first pathway involved by hypomethylated genes, which might be due to the selection of kidney cell lines. The kidney mainly maintained water and acid–base balance through vasopressin-regulated water reabsorption pathway and proximal tubular reabsorption pathway (Luo et al., 2022). PRV infection broke this balance relationship and the m^6^A modification level of related genes decreased. Hypomethylated genes were also enriched in NF-κB signaling pathway. PRV triggered the activation of NF-κB signaling pathway through DNA damage response, and viral late factors could effectively inhibit NF-κB-dependent genes expression (Romero and Favoreel, 2021), which might also be regulated by m^6^A modification. PRV infection might regulate the expression of these cytokines by changing m^6^A modification, and the specific mechanism needed further study. Hypermethylated genes were mainly involved in spliceosome and neurological disorders pathways. HSV-1 has been proved to be a suspected cause of AD and Amyotrophic lateral sclerosis (ALS; Dash et al., 2020; Ge and Yuan, 2022). Moreover, PRV could invade the nervous system (Papageorgiou et al., 2022), so m^6^A modification might regulate the invasion of PRV. PRV relieved or strengthened the regulation of related pathways by regulating m^6^A modification, thereby facilitating virus lysis and replication. The m^6^A modification level of total RNA decreased in PRV-infected PK15 cells at 24 hpi. Coincidentally, HSV-1 could reduce the m^6^A modification level after infecting host cells (Srinivas et al., 2021). Quantitative detection of the m^6^A modification level in all mRNA after infection with PRV or HSV-1 by mass spectrometry was also decreased (Jansens et al., 2022). PRV infection significantly reduced the m^6^A modification level of host RNAs. Overall, PRV infection affected the cellular m^6^A transcripts to a certain extent.

The common m^6^A modification motifs were detected by DREME to determine whether m^6^A peaks had common sequences that potentially specify methylation. Specific motifs changed slightly in PRV-infected PK15 cells. The change of m^6^A regulators activity caused by infection might alter the recognition rate of motifs, thereby causing the change of the specific base content of motifs. The most common motif in the m^6^A peaks of PRV transcripts was UCRU. Although the most common motif was not the traditional DRACH (G/A-G/A-A-C-A/C/U), there were specific m^6^A modification consensus motifs in *E*. *coli* (Deng et al., 2015), *A*. *thaliana* (Wan et al., 2015), duckling liver (Wu et al., 2022), and the AC-containing motif was also not found in the common m^6^A motif in human embryonic kidney cells (HEK293; Li et al., 2019), indicating that the most common m^6^A motif varied among different species, even in different cell lines of the same species.

Currently, PRV itself has not been found to encode m^6^A regulators, so PRV infection may regulate m^6^A modification level by altering the cellular expression of m^6^A regulators. The mRNA and protein expression levels of ALKBH5 decreased rapidly after PRV infection, which indicated that ALKBH5 was inhibited immediately, thereby facilitating rapid viral replication, meanwhile ALKBH5 was the major demethylase involved in the regulation of PRV replication. After 24 hpi, both mRNA and protein levels of m^6^A regulators except YTHDF3 were significantly decreased, which was similar to many alphaherpesvirus that globally impair host mRNA stability and ongoing protein synthesis (Sandri-Goldin, 1994; Everly et al., 2002; Walsh and Mohr, 2011; Sciabica et al., 2014). Moreover, the expression of US3 of alphaherpesvirus was the cause of inactivation of methyltransferase complex (Jansens et al., 2022). Miraculously, the mRNA level of YTHDF3 was increased at 12 hpi, but its protein expression level decreased significantly. Nevertheless, the mRNA level of YTHDF3 was significantly decreased at 24 hpi, but its protein expression level was equivalent to that of the control group without virus infection. The differential outcomes of YTHDF3 mRNA and protein expression levels caused by PRV infection and the related translational regulatory mechanisms need to be further investigated. After HSV-1 infected different cells, the mRNA levels of m^6^A specific binding proteins changed inconsistently (Feng et al., 2021), indicating that different cell types would also lead to differences in regulation patterns. PRV infection might affect mRNA translation, alternative splicing and regulate the stability of host proteins expression by altering the distribution pattern of m^6^A modification.

Pseudorabies virus infection caused alterations in host m^6^A modifications, we then probed the effects of host m^6^A regulators on viral replication. Among RNA viruses, knockdown of YTHDF2 significantly increased Zika virus (ZIKV) replication (Lichinchi et al., 2016), and knockdown of METTL3/14 promoted the production of hepatitis C virus (HCV) particles (Gokhale et al., 2016). The replication of DNA viruses such as Simian vacuolating virus 40 (SV40; Tsai et al., 2018) and Bombyx mori nucleopolyhedrovirus (BmNPV; Zhang et al., 2020) were also regulated by m^6^A regulators. Our study found that knockdown of methyltransferases METTL3/14 in PK15 cells inhibited the replication of PRV (Figures 4, 5), while knockdown of demethylase ALKBH5 significantly promoted PRV proliferation, and the regulation of FTO was not obvious (Figure 6). Knockdown of the specific binding proteins YTHDF2/3 decreased the replication of PRV, but knockdown of YTHDF1 had no effect (Figure 7). YTHDF1/2/3 had the same effect on some virus replication, such as simultaneously promoting human respiratory syncytial virus (HRSV) replication (Xue et al., 2019) or inhibiting HCV infection (Gokhale et al., 2016). However, for EBV proteins, YTHDF1 increased their expression, while YTHDF2/3 inhibited (Zheng et al., 2021). The regulation of YTHDF1/2/3 on human immunodeficiency virus I (HIV-1) was also different in different cell lines (Kennedy et al., 2016; Lu et al., 2018). YTHDF1 promoted translation of m^6^A-modified mRNAs (Wang et al., 2015). YTHDF2 promoted m^6^A-modified mRNAs degradation (Du et al., 2016). YTHDF3 functioned both as YTHDF1 promoted translation and YTHDF2 promoted degradation of mRNAs (Li et al., 2017; Shi et al., 2017). Meanwhile, YTHDF1/2/3 mediated the degradation of mRNAs (Zaccara and Jaffrey, 2020). These may explain why studies involving YTHDFs often show conflicting results. In PRV-infected cells, whether YTHDFs can directly recognize viral mRNA to play a regulatory role and the interaction of m^6^A regulators with PRV-specific proteins remains to be further elucidated.

Pseudorabies is a related disease caused by PRV infection, and to date, there are no fully effective treatments or treatments are insufficient, and exploring potential targets for antiviral therapy may provide new therapeutic directions. Epigenetic modifications on RNA have been found to be associated with a variety of diseases (Chen et al., 2018; Su et al., 2020; Yankova et al., 2021). Since the presences of m^6^A are involved in multiple viral life cycles, the development of inhibitors targeting m^6^A regulators and m^6^A modification level can serve as novel therapeutic means achieving antiviral effects. The most widely studied m^6^A modification inhibitor was 3-DAA, which inhibited the m^6^A modification level by depleting the level of S-adenosylmethionine (SAM) in cells without affecting the capping of mRNA (Bader et al., 1978), and could effectively inhibit the replication of viruses such as KSHV (Fustin et al., 2013; Ye et al., 2017), HSV-1 (Feng et al., 2021), enterovirus 71 (EV71; Yao et al., 2020). 3-DAA not only effectively inhibited the virus *in vitro*, but also inhibited the replication of multiple viruses in mice and rats (Bader et al., 1978; Wyde et al., 1990; Bray et al., 2000; Kennedy et al., 2016; Courtney et al., 2017). Therefore, we selected 3-DAA as the inhibitor of m^6^A modification to investigate the effect of m^6^A modification level on PRV replication. With the increase of 3-DAA concentration, the CPE caused by PRV decreased significantly, the viral titers, copies and gE protein expression decreased with the increase of 3-DAA concentration in a dose-dependent manner (Figure 8). Although it could not be explained that the inhibition of m^6^A modification production by 3-DAA in cultured cell lines was the only inhibitory mechanism to prevent PRV replication, it could be shown that drugs that reduce the production of m^6^A modification could effectively inhibit PRV replication. In addition, since 3-DAA inhibits all types of RNA methylations, it is difficult to determine whether this antiviral effect comes from inhibiting the methylation of mRNA cap structure or from mRNA internal methylation. Therefore, 3-DAA is not a drug that specifically inhibits the m^6^A modification level. Meclofenamic acid (MA), an inhibitor of demethylase FTO, could enhance the expression of KSHV cleavage genes (Ye et al., 2017). In-depth exploration of m^6^A regulators specific inhibitors can be used as a new research direction of antiviral targeted drugs, which also helps to study the molecular mechanism of virus replication. For anti-PRV infection, we can focus on the development of m^6^A modification level inhibitors, methyltransferase inhibitors or demethylase enhancers as targeted drugs.

**Figure 9:**
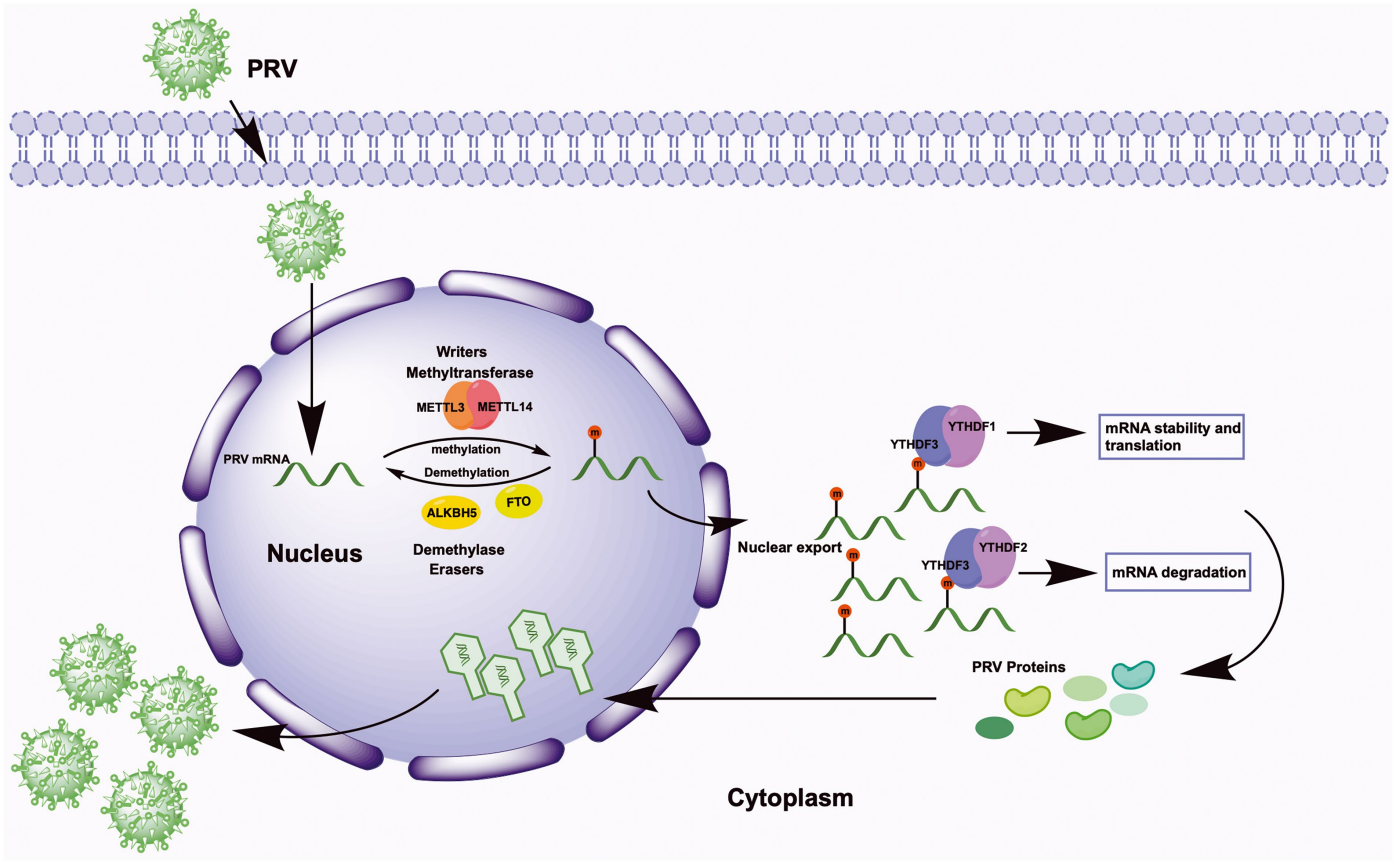
Schematic representation of m^6^A regulation of PRV replication. Upon viral infection, virions first attach to the host cell surface, subsequently enter the cell, and finally the viral genome is released into the host cell nucleus. In the nucleus, the methyltransferases METTL3/14 co-induce the methylation of multiple viral mRNAs, whereas the demethylases FTO and ALKBH5 regulate the demethylation process. The methylation of viral mRNA promotes its own nuclear export. In the cytoplasm, YTHDF1 and YTHDF3 synergistically promote mRNA stability and translation, and YTHDF3 cooperates with YTHDF2 to promote mRNA degradation. Ultimately, the expression of PRV proteins is promoted by the cooperation of YTHDF1/2/3, and these products are transported back into the nucleus, where they complete the viral nucleocapsid assembly and eventually release more viral particles.

In conclusion, we explored the m^6^A modification sites in PRV transcripts cultured in PK15 cells for the first time. We provided evidence that m^6^A modification contributes to the reciprocal regulation of PRV and host, and m^6^A modification could promote PRV infection as a positive regulatory effect (Figure 9). The host m^6^A modification map also changed significantly during PRV infection, which was beneficial for understanding the mechanism of PRV-host interactions. m^6^A modification as a conservative target, this work emphasized the possibility of developing m^6^A modification inhibitors as anti-PRV infection in the future.

## Data availability statement

The datasets presented in this study can be found in online repositories. The names of the repository/repositories and accession number(s) can be found at: https://www.ncbi.nlm.nih.gov/geo/, GSE209949.

## Ethics statement

The animal study was reviewed and approved by the Committee of Experiment Operational Guidelines and Animal Welfare of Sichuan Agricultural University.

## Author contributions

Q-GY provided the initial idea and contributed to the conception of this article. P-LY conceived and designed the study and wrote the manuscript. RW, S-JC, Y-PW, X-BH, SZ, Y-FL, S-YD, J-CL, S-MY and QZ analyzed the data. All authors read and approved the final manuscript.

## Funding

This work was supported by the Sichuan Science and Technology Program, China (Grant no. 2020YFS0011) and Natural Science Foundation of Sichuan Province, China (Grant no. 2022NSFSC1692 and 2022NSFSC1625).

## Conflict of interest

The authors declare the research was conducted in the absence of any commercial or financial relationships that could be construed as a potential conflict of interest.

## Publisher’s note

All claims expressed in this article are solely those of the authors and do not necessarily represent those of their affiliated organizations, or those of the publisher, the editors and the reviewers. Any product that may be evaluated in this article, or claim that may be made by its manufacturer, is not guaranteed or endorsed by the publisher.
