# Supplementary material for: Pseudorabies virus exploits N6-methyladenosine modification to promote viral replication
Source: Front Microbiol. 2023 Feb 3;14:1087484. doi: 10.3389/fmicb.2023.1087484 (PMC9936159; doi:10.3389/fmicb.2023.1087484)
Supplement: Supplementary file 1 [file Data_Sheet_1.docx]

Supplementary Material

# Supplementary Figures and Tables

## Supplementary Figures


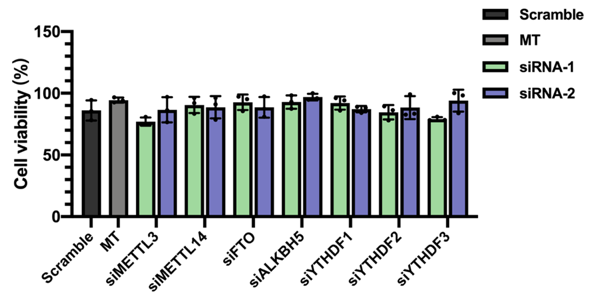


**Supplementary Figure 1.** MTT assay for cell viability after transfection of siRNAs for 48h (n = 3).


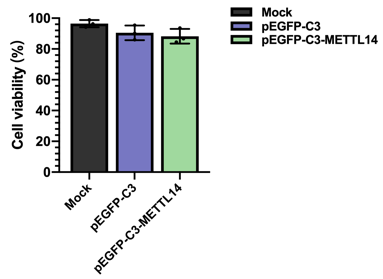


**Supplementary Figure 2.** MTT assay for cell viability after transfection of pEGFP-C3-METTL14 and pEGFP-C3 (n = 3).


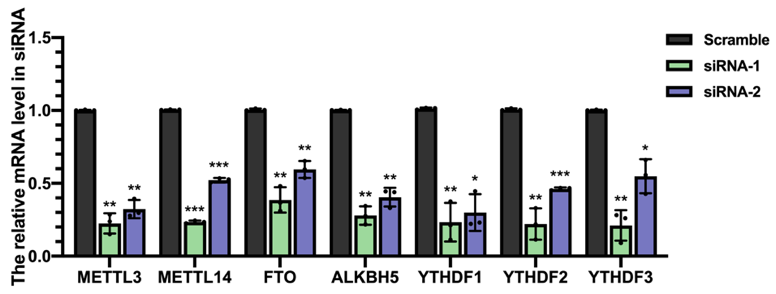


**Supplementary Figure 3.** Knockdown efficiency of siRNAs for m6A regulators. The mRNA levels of m6A regulators were determined by RT-qPCR (n = 3). *p<0.05, **p<0.01, ***p<0.001.


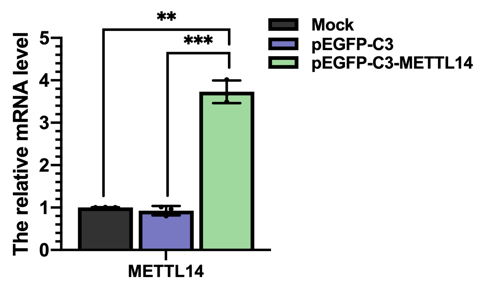


**Supplementary Figure 4.** The relative mRNA level of METTL14. The mRNA levels of METTL14 were determined by RT-qPCR (n = 3). **p<0.01, ***p<0.001.

**
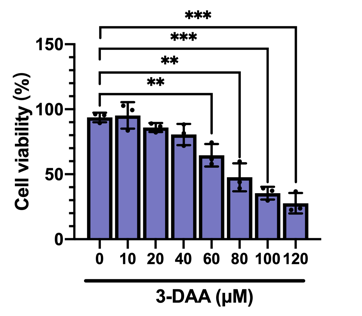
**

**Supplementary Figure 5.** MTT assay for cell viability after treated with 3-DAA (n = 3). **p<0.01, ***p<0.001.

## Supplementary Tables

| Target name | Sense (5’-3’) | Antisense (5’-3’) |
| --- | --- | --- |
| siMETTL3-1 (100513294-937) | CAACAGAGCAAGAAGGUUATT | UAACCUUCUUGCUCUGUUGTT |
| siMETTL3-2 (100513294-1191) | CCACAUGGAUACCUGCAAATT | UUUGCAGGUAUCCAUGUGGTT |
| siMETTL14-1 (100525761-293) | AAAGAUGAGCAGAGAGAAATT | UUUCUCUCUGCUCAUCUUUTT |
| siMETTL14-2 (100525761-1943) | GCCACUGCUAUCACAGCAATT | UUGCUGUGAUAGCAGUGGCTT |
| siFTO-1 (100127165-230) | GCACAAGCAUGGCUGCUUATT | UAAGCAGCCAUGCUUGUGCTT |
| siFTO-2 (100127165-1260) | GCUGUGCUUCGUGAAGUUATT | UAACUUCACGAAGCACAGCTT |
| siALKBH5-1 (100622998-652) | GCUGCAAGUUCCAGUUCAATT | UUGAACUGGAACUUGCAGCTT |
| siALKBH5-2 (100622998-1548) | CCUCAGGAAGACAAGGUUATT | UAACCUUGUCUUCCUGAGGTT |
| siYTHDF1-1 (110257488-181) | GCGUUCAUGACAGUGACUUTT | AAGUCACUGUCAUGAACGCTT |
| siYTHDF1-2 (110257488-1036) | GCAACAGAAACGCGGCAUUTT | AAUGCCGCGUUUCUGUUGCTT |
| siYTHDF2-1 (100623159-407) | CCGGCUUUAAUUGGUGUUUTT | AAACACCAAUUAAAGCCGGTT |
| siYTHDF2-2 (100623159-2178) | ACGUCAAGGUCGUGGGAAATT | UUUCCCACGACCUUGACGUTT |
| siYTHDF3-1 (106510070-127) | GAGGAAACAGGCGAAGAAUTT | AUUCUUCGCCUGUUUCCUCTT |
| siYTHDF3-2 (106510070-1833) | GGAGAGAAAUAGAAACAAATT | UUUGUUUCUAUUUCUCUCCTT |
| siNC | UUCUCCGAACGUGUCACGUTT | ACGUGACACGUUCGGAGAATT |

**Supplementary Table 1.** siRNA sequences.

| Gene name | Forward primers (5’-3’) | Reverse primers (5’-3’) |
| --- | --- | --- |
| PRV *gB* | CTGGTGACCTTCGAGCACAA | CTCGTAGTACACGTACCCGC |
| porcine *METTL3* | ATCGTAGCCGAGGTTCGTTC | GTTGCACATTGTGTGGTCGT |
| porcine *METTL14* | GTGTGTTTACGCAAGTGGGG | TCCCATAAGGCAGTGTTCCTTT |
| porcine *FTO* | AGAAATCCCGATGTGTGGCA | GGCATTGAGATCATCCAGCAT |
| porcine *ALKBH5* | CGTGACTGTGCTCAGTGGATATG | ACCGAGGCGCATCTAACCTT |
| porcine *YTHDF1* | TGGACCCCCAGAGAACGA | TGGTTTGACTGTCCAGAAAGGT |
| porcine *YTHDF2* | GCAGGTGTTGAAAATCATAGCC | CACGACCTTGACGTTCCTTTTT |
| porcine *YTHDF3* | GGGCAAGGAAATAAAGGTGGGA | TAGAACATGGGCCTTAGGAGC |
| porcine *GAPDH* | CGTGTCGGTTGTGGATCTGA | CTCAGTGTAGCCCAGGATGC |

**Supplementary Table 2.** The primers were used for RT-qPCR analysis.
